# Esthetic Perception of Different Clinical Situations of Maxillary Lateral Incisor Agenesis According to Populations with Dental and Non-Dental Backgrounds: A Systematic Review and Meta-Analysis

**DOI:** 10.3390/dj11040105

**Published:** 2023-04-17

**Authors:** Maria João Calheiros-Lobo, Mafalda Calheiros-Lobo, Teresa Pinho

**Affiliations:** 1 UNIPRO-Oral Pathology and Rehabilitation Research Unit, University Institute of Health Sciences (IUCS), Cooperativa de Ensino Superior Politécnico e Universitário (CESPU), Rua Central de Gandra 1317, 4585-116 Gandra, Portugal; 2 Conservative Dentistry, Department of Dental Sciences, University Institute of Health Sciences (IUCS), Cooperativa de Ensino Superior Politécnico e Universitário (CESPU), Rua Central de Gandra 1317, 4585-116 Gandra, Portugal; 3 Pediatrics Dentistry and Orthodontics, Department of Dental Sciences, University Institute of Health Sciences (IUCS), Cooperativa de Ensino Superior Politécnico e Universitário (CESPU), Rua Central de Gandra 1317, 4585-116 Gandra, Portugal; 4 IBMC-Institute for Molecular and Cell Biology (IBMC), Institute of Innovation and Investigation in Health (i3S), University of Porto, R. Alfredo Allen, 4200-135 Porto, Portugal

**Keywords:** maxillary lateral incisor agenesis, esthetic perception, laypersons, general dentist, dental professional, orthodontist

## Abstract

Treatment of unilateral or bilateral maxillary lateral incisor agenesis is challenging, time-consuming, expensive, and requires careful treatment planning, predictability, and esthetics. This review aimed to identify differences in esthetic perception among orthodontists, general dentists, differentiated dentists, and laypersons, which may interfere with treatment options. EBSCO, PubMed, ScienceDirect, Cochrane Library databases, and Google Scholar were searched using keyword pairing and a Boolean expression, “(congenitally missing OR agenesis OR hypodontia) AND (maxillary lateral incisors) AND (esthetic perception OR smile) AND (laypersons OR dental professional OR general dentist OR orthodontists).” Reviews and case studies were excluded. A total of 13 studies were selected for qualitative analysis (adapted ROBINS-I) and 11 were selected for meta-analysis (*p* < 0.05) after being sub-grouped into “Opening vs. Closure” and “No remodeling vs. Dental remodeling vs. Dental and gingival remodeling” groups. A meta-analysis evaluated the magnitude of the difference between groups based on differences in means and effect sizes (α = 0.05; 95% CI; Z-value 1.96), revealing that the esthetic perception of maxillary lateral incisor agenesis treatment remains controversial even among professionals. Gingival remodeling was not valued compared to isolated dental remodeling. Studies lack rigorously comparable methodologies. Discussion with the patient is pertinent in doubtful situations, as the best treatment option remains unclear, and overtreatment should be avoided.

## 1. Introduction

Esthetic perception of the smile involves a subjective response to visual sensory stimuli and the ability to recognize and appreciate qualities such as symmetry, balance, proportion, and harmony [1,2]. It is a complex cognitive and emotional process involving both conscious and unconscious parts of the mind and is influenced by factors such as culture, personal experiences, and context, meaning that different individuals may have different esthetic preferences and evaluations [3,4]. A balanced, symmetrical smile is considered essential for facial esthetics, influencing facial expression, overall physical appearance, emotional expression, individual personality, and psychological well-being [5]. Agenesis (developmental absence) of anterior teeth negatively affects interpersonal relationships and self-esteem, leading patients to seek treatment [6,7,8,9]. A patient’s self-image and expectations play an essential role in clinical treatment decisions [10,11] similar to the esthetic judgment of dentists [12,13].

Maxillary lateral incisor agenesis (MLIA) is the second most frequent kind of non-syndromic congenital tooth agenesis with a 1–2% prevalence in the Caucasian population, being bilateral in more than half of cases, and if unilateral, frequently is associated with peg-shaped laterals on the contralateral side [6,14,15,16,17].

Treating unilateral or bilateral MLIA is challenging, involving space opening (SOP) with subsequent prosthetic replacement of the missing lateral incisor or space closure (SCR) by moving the canine into the edentulous space followed by tooth remodeling (porcelain crown or resin-matrix composite restorations) for a complete tooth match with the contralateral incisor [6,7,9,14,16,18,19]. Both treatment options are expensive, time-consuming, complex, and controversial [16,20,21,22,23], but unless extractions are made, no significant differences exist between them concerning the time spent in treatment [24].

SCR often requires remodeling of the canine into a lateral incisor and of the first premolar into a canine to match the anatomy, color, and gingival contour of a naturally erupted tooth [6,7,16,25]. A post-treatment periodontal evaluation over a period from 6 months to 7 years found no significant differences in plaque index and bleeding index between the SCR and SOP groups [26], in contradiction with findings from other cohorts wherein SCR patients possessed better periodontal health after 5 years post-treatment [22,27]. A thick periodontal biotype was associated with the SOP group, and the thin biotype was associated with the SCR and control groups [26]. Patients with MLIA treated with space closure, first premolar intrusion, and canine extrusion were found periodontally healthy 10 years after treatment with a periodontal status comparable with the condition of patients without MLIA who have received similar orthodontic treatment [28].

Concerns to take into account include the possibility of root resorptions in cases involving orthodontic treatment [11] and the level of gingival exposure during smiles in cases involving lateral incisor substitutions with an implant-supported crown [29]. Despite the absolute position of the gingival zeniths, clinical situations treated with implants show values relative to the reference line, similar to those of aligned teeth without lateral incisor agenesis [30,31].

Perception and esthetic-judgment studies can help dental professionals understand how laypersons evaluate all the details concerning their smiles and those of others and prioritize patients’ needs to avoid biased professional evaluations [7,8,22,32]. Concerning the esthetic perception, compared to general dentists and orthodontists, laypersons tend to accept a larger scale of smile deviations, such as midline deviation up to 2.2 mm, exposed gingival margins while smiling, and variation in tooth coronal morphology [8,16,33,34], perhaps because laypersons observe the eyes in the images before attending the mouth [35]. 

Esthetic results play an essential role in managing the clinical situation of MLIA, and general dentists and orthodontists tend to overestimate their importance more than their functional aspects [16]. However, each professional has a fickle opinion when asked to choose the best treatment to follow (closure or space opening) or about the esthetic result obtained in cases already treated [36], and the education levels of professionals’ dental backgrounds also seem to play a role [4,37]. Back in 1976, Senty [38] said that the “diagnostic decision to open or close the space is always a compromise” and that one simple question is to be answered: “Which is the best compromise for the patient taking an interest both functional and esthetic?”

Since then, despite evolution in technological and professional technical quality, doubts persist [39], mainly when treating young patients, especially in unilateral situations of MLIA, as subjects treated with implant-supported crowns will have inevitable long-term infraocclusion of the replaced lateral incisors [27] or will experience the need for periodic maintenance if treated with conservative restorative techniques [20,27,39,40,41].

A systematic review to elucidate the esthetic perception of laypersons, general dentists, and orthodontists in MLIA clinical situations may help evidence-based treatment decisions, especially in doubtful clinical situations in which any treatment option is valid.

The primary aim of this systematic review was to clarify the differences in esthetic perception between populations with dental or non-dental backgrounds and to compare the esthetic perception of MLIA situations treated with space closure with those treated with space opening. In space-closure situations, determining the esthetic impact of dental and gingival remodeling of the mesialized canine, with or without symmetry, was also considered pertinent. The authors hypothesized that esthetic perception would be the same among all observers when evaluating the treated clinical situations of MLIA.

## 2. Materials and Methods

### 2.1. General Aspects

The review followed the preferred reporting items for systematic reviews and meta-analysis (PRISMA) 2020 recommendations [42]. The population, intervention, comparison, and outcome (PICO) question was: “Which of the treatment options, closure or space opening, whether MLIA is unilateral or bilateral, is perceived as more esthetic by different populations?” The clinical situation of the treated MLIA constituted the study population. The treatment option was intervention, specifically the closure or opening of the space. A comparison was made between unilateral and bilateral situations, and between canine approaches performed by the dentist. The outcome was esthetic scoring by observers (laypersons, patients, general dentists, orthodontists, and other dental professionals).

### 2.2. Search Strategy and Criteria

Electronic databases (EBSCO, Medline/PubMed, ScienceDirect, and Cochrane) and the search engine Google Scholar were searched from 1 January 2000 to 31 July 2022 by pairing the keywords congenitally missing, maxillary incisors lateral, anterior tooth agenesis, agenesis, hypodontia, esthetic, aesthetic, perception, smile, laypersons, dentist general, dental professional, orthodontists, and by the Boolean expression: “(congenitally missing OR agenesis OR hypodontia) AND (maxillary lateral incisors) AND (esthetic perception OR smile) AND (laypersons OR dental professional OR general dentist OR orthodontists).” Articles written in languages other than English or Portuguese, literature or systematic review articles, and case studies were excluded. To frame the universe of publications related to the theme and validate the chosen Boolean expression, an open search independently combining keywords was performed in previous databases and the Google Scholar search engine [43] followed by filtering within the methodology.

Three investigators (M.J.C.L., M.C.L. and T.P.) discussed the search strategy. Articles identified via the search strategy, following the exclusion criteria, and once-set concordant standards were independently selected by two researchers (M.J.C.L. and M.C.L.) after removing duplicates. Publications and titles were analyzed followed by abstract reading and a complete analysis of the selected articles. The references in the selected papers were subjected to a detailed search for potentially relevant articles.

### 2.3. Data Extraction and Collection

Data on the esthetic perceptions of professionals or laypersons, types of treatment, and symmetries of procedures were extracted from the selected studies and organized in tables. Disagreements between the reviewers in these two stages were resolved by consensus with a third researcher (T.P.). 

To better understand and interpret the results and methodologies, the authors formed a group comparing procedures involving opening the space to procedures involving the closure of the space (“Opening vs. Closure”) and another within the space-closure group that compared the type of canine approach performed by the dentist (“Canine without remodeling vs. Canine with dental remodeling vs. Canine with dental and gingival remodeling”). For convenience and comparison, the scale of 0–100 mm was converted to a 0–10 scale. Mean conversion with a 95% confidence interval (*p* < 0.05) was performed to standardize the results of different studies using a previously described methodology [44,45]. The sample size for each study is presented in a short table.

### 2.4. Methodological Quality

An adapted methodological quality analysis using seven items based on the risk of bias in non-randomized studies of interventions (ROBINS-I) [46] assessed the risk of bias in the selected studies. Parameters considered essential for risk of bias assessment were adapted as previously described in other dentistry studies [47]. The sample size calculation, accurate description of the sample, occurrence of dropouts, use of valid methods, presence of confounding variables, blind measurements, proper statistical analysis, and final overall assessment of the articles were used. Each study was scored as High (5–7), Moderate (3–4), or Low (0–2) quality. 

The sample-size calculation established the number of individuals included to obtain valid conclusions. A sample description was considered correct if the origin and main features of the sample were described and if the degree of professional specialization was described in those cases involving health professionals. The presence of dropouts was a missing item because the participants were voluntarily committed to responding to the survey after visualizing the clinical situations presented and were not involved in studying the technique. The employed method was evaluated using valid method parameters. The presence of confounding variables analyzed aspects related to the model used, which could confuse or abstract participants from the crucial study details. 

The “blind measurement” parameter implies the unknowledge of the cases to be assessed for qualitative or quantitative evaluations, avoiding the usurpation of opinions. Correct descriptive and inferential statistics were analyzed using appropriate statistical analysis parameters. 

### 2.5. Meta-Analysis

A meta-analysis focusing on the esthetic evaluations of treatment options according to the observers was conducted using a software program (Stata v17.0; StataCorp, Lakeway, TX, USA). Subgroup analyses were conducted according to author, type of treatment, unilateral or bilateral MLIA, and type of recontouring (canine or gingival interventions). 

Statistical heterogeneity was determined using the I^2^ test (α = 0.05). A random-effects meta-analysis model with restricted maximum likelihood was used to compare the means across all studies (*p* < 0.05). Subgroups with studies that provided control images were formed to determine intra- and inter-study heterogeneity after calculating the difference between means and effect sizes (α = 0.05; 95% CI; Z-value 1.96). The Hedge’s g statistic was preferred to be more adequate for small samples and significantly different sample sizes. Funnel and Galbraith plots were used to assess publication bias (random-effects model; α = 0.05; 95% CI; Z-value 1.96) and heterogeneity (random-effects model; α = 0.05; 95% CI; Z-value 1.96). A paired *t*-test (*p* < 0.05; 95% CI) was run for subgroups to determine whether there was a statistically significant mean difference between the initial image and the remodulation image after analyzing data for normality (Shapiro–Wilk test of normality, *p* < 0.05; 95% CI) and the identification of significant outliers (boxplot, *p* < 0.05; 95% CI), assuming that the variables were continuous and the groups were related. In the absence of a control image, data obtained for the “No remodeling” group were considered control references for comparison with the “Dental remodeling” or “Dental and gingival remodeling” groups.

Two studies selected for data analysis were not plotted because one [6] reported a qualitative rating, and the other [19] did not rate the images independently but as a synoptic global evaluation, not distinguishing bilateral from unilateral situations, and using a numerical scale impossible to convert for quantitative analysis.

## 3. Results

### 3.1. Study Selection

The search of the different databases with a Boolean expression and after duplicate removal retrieved 36 articles, of which 6 were excluded due to their titles, 10 were excluded due to their abstracts, and 7 were excluded after integral reading revealed that they failed to meet the set objectives or were not related to MLIA. Finally, one [48] was added through a manual search. A total of 13 studies [6,7,9,14,16,18,19,25,48,49,50,51,52] were included in the qualitative analysis. 

**Figure 1:**
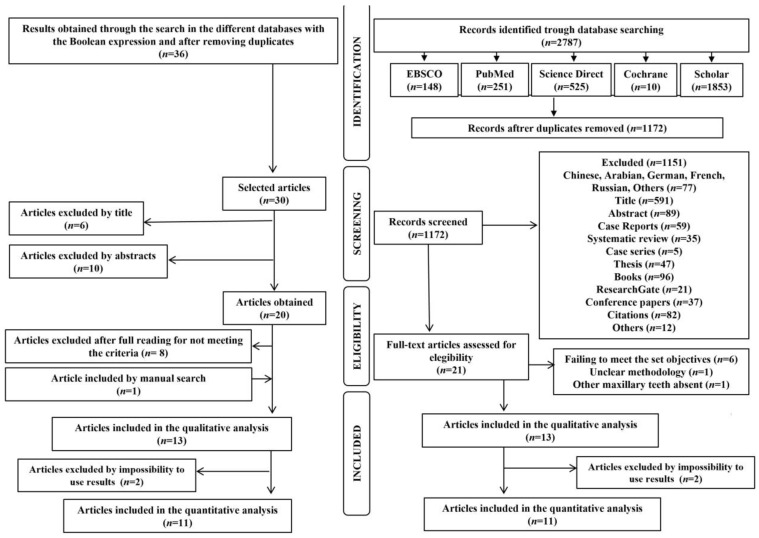
Synopsis of the PRISMA strategy for focused and broad article selection. (EBSCO, EBSCO-Information Services, library database services (electronic databases, e-books, and other library resources); PUBMED, free web search engine; accessing primarily the MEDLINE database of references and abstracts on life sciences and biomedical topics; Scholar, Google Scholar, free web search engine, index of full text or metadata of scholarly literature across an array of publishing formats and disciplines).

The broad search strategy using keyword pairing retrieved 2787 titles. After duplicate removal and post-search filter application (language, type of publication, study objectives, and no-MLIA), the same studies attained through the Boolean search remained, including the one that was manually introduced. The selection strategy is illustrated in Figure 1.

### 3.2. Study Characteristics and Descriptive Data Analysis

The methodological quality analysis is summarized in Table 1.

Only three studies [9,14,16] used sample size calculations to confirm the inclusion of sufficient individuals to represent the population. Concerning the correct sample description, globally, there was a lack of distinction between general dentists and specialists, namely orthodontists, not always well-defining their professional training and not being, in some cases, officially considered specialists [16,18,19,48,52]. The digital model [14,16,48] and the real model [6,7,9,18,19,25,49,50,51,52] were considered valid for meeting the goals proposed by the authors. 

Blind measurements were specified in only one study [19]. The item “opening vs. closure” was present in three studies [18,49,50] comparing bilateral opening with bilateral closure and one study [7] comparing unilateral opening with bilateral closure (with dental remodeling). In the space-closure group, data to distinguish unilateral from bilateral situations from nine studies [7,9,14,16,18,25,48,51,52] were registered along with data from a comparison of canines without remodeling, with dental remodeling only, or canines with dental and gingival remodeling. 

The results of the analyzed studies were acquired, presented, and grouped based on different classification scales. In most cases, participants performed both quantitative and qualitative evaluations. Numerical ranges were found from 0 to 10 ((1)-less attractive and (10)-more attractive) and from 0 to 100 mm (VAS analogic scale) ((0–50.99 mm) -unpleasant and (>51 mm)-pleasant). Studies using the 0–100 mm scale showed greater dispersion values, unlike those using the 0–10 scale, assuming only whole numbers. 

Table 2 shows the sample size found in the analyzed studies, revealing heterogeneous observers in terms of type and number.

Table 3 presents the data extracted from the selected studies. The 13 studies submitted for quality analysis were non-randomized, and 5 studies [6,16,18,48,49] did not include control images.

The two studies that are not plotted are briefly summarized here, considering their relevance to a broader discussion. The first study [6] compared the attractiveness of smiles between patients with MLIA and those with complete natural dentition. In general, maxillary canine morphology was perceived by orthodontists, dentists, and laypersons. Broad canines were classified as unattractive, and narrower canines were classified as more attractive in all groups. Sharp canines were rated negatively by all the groups. The second study [19] compared the esthetic attractiveness of adhesive Maryland bridges, implant-supported crowns, canine mesialization, and natural dentition without MLIA, based on a series of dental photographs from MLIA clinical situations. There was no agreement among the dental professional groups or between these groups and laypersons regarding the best score for space closure with canine mesialization. Implant-supported crown substitution after space opening had the highest score, indicating less attractiveness, as higher scores reflected a less-favorable evaluation (best score (7) and worst score (35)). Assessments of symmetrical and asymmetrical clinical situations were combined, making it impossible to obtain references to the influence of symmetry or asymmetry on smile perception. 

### 3.3. Meta-Analysis

**Figure 2:**
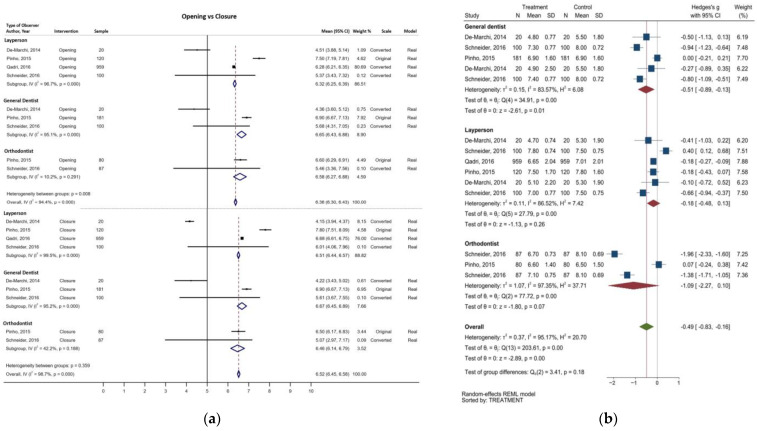
Forest plots summarizing results of group “Opening vs. Closure.” (**a**) Comparison according to author and observer. (**b**) Difference in means with Hedges’s g effect between control and intervention groups with data obtained from the included studies [7,18,49,50].

**Figure 3:**
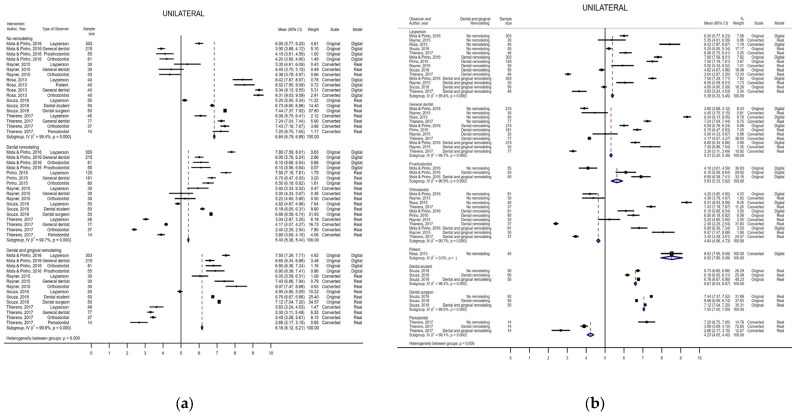
Forest plot summarizing results obtained in group “No remodeling vs. dental remodeling vs. dental and gingival remodeling”, UNILATERAL. (**a**) Comparison according to type of remodeling by the same author. (**b**) Comparison according to observer and type of remodeling, with data obtained from the included studies [9,14,16,18,51].

**Figure 4:**
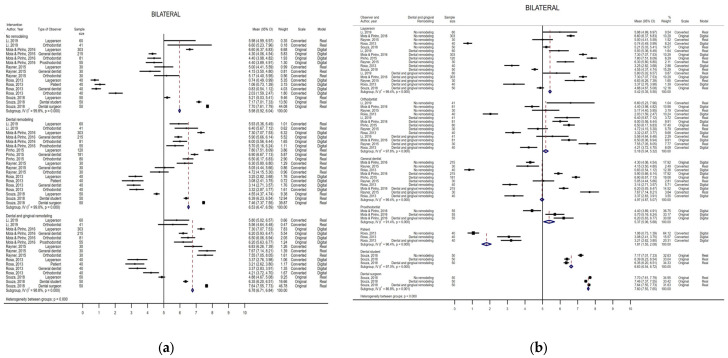
Forest plot summarizing results obtained in group “Canine without remodeling vs. Canine with dental remodeling vs. Canine with dental and gingival remodeling”, BILATERAL. (**a**) Comparison according to type of remodeling by the same author. (**b**) Comparison according to observer and type of remodeling, with data obtained from the included studies [9,14,16,18,48,51].

A comparative analysis of the data collected from the 11 studies available for quantitative analysis is shown in Figure 2, Figure 3 and Figure 4. Data on the calculation of the difference in means and effect size (α = 0.05; 95% CI; Z-value 1.96) are presented in Supplementary Table S1. Forest plots with differences in means, by author and (a) type of remodeling, (b) type of agenesis, and (c) observer can be found in Supplementary Figure S1a–c.

When comparing opening and closure (Figure 2a), the data showed no significant differences (*p* < 0.05) between the two treatment types in the four studies analyzed [7,18,49,50]. These forest plots highlight the relatively low scores observed for both treatment options in the study by De-Marchi et al. [7], a high dispersion of values in the study by Schneider et al. [50], and a trend toward higher scores according to laypersons compared to dental professionals in the study by Pinho et al. [18]. The study by Qadri et al. [49], which had the largest sample, showed no difference between opening or space closure for the same observer type. 

When analyzing the difference in means and effect size (α = 0.05; 95% CI; Z-value 1.96) (Figure 2b), the magnitude of the overall effect was medium (g = 0.5; *p* < 0.05) for the intervention with larger variations occurring in the study by Schneider et al. [50] in values according to orthodontists. 

Overall, in unilateral MLIA, as shown in Figure 3a,b, there was a slight decrease in the scoring of smiles between no remodeling and dental and gingival remodeling. When comparing results within the same article, the study by Rayner et al. [9] showed an increase in rating with increased canine reshaping in all categories of participants, wherein laypersons presented higher assessments than those of professionals, except for dental and gingival remodeling, wherein professionals considered this kind of procedure far more esthetic. Mota and Pinho [14] presented a more significant score increase between the canine without remodeling and the canine with dental remodeling, and the lowest increase for the canine with the two types of remodeling (*p* < 0.05). In this study, the scoring from laypersons was higher than that of professionals for all types of remodeling, appearing less pronounced for complete remodeling (*p* < 0.05). Souza et al. [51] showed a tendency for worse scores for dental remodeling only compared to other interventions in all groups of observers with laypersons presenting the lowest values.

By analyzing the studies that included only one type of procedure, Rosa et al. [16] revealed lower esthetic results in all groups of participants in the case of canines without remodeling, whereas Pinho et al. [18] (canines with dental remodeling) found values similar to those of other studies using the same procedure. Data from Thierens et al. [52] showed lower scores for dental only or dental and gingival remodeling compared to no remodeling and high heterogeneity among groups of observers. 

If the MLIA was bilateral (Figure 4a,b) with symmetry of the smile, there was no significant discrepancy in esthetic appreciation (*p* < 0.05); however, there was a more positive assessment regarding the groups with dental and gingival remodeling. As an exception, the study by Rosa et al. [16] showed a negative appreciation for all types of remodeling according to all observers.

When comparing the different types of procedures within each article, in the study by Rayner et al. [9], unlike what happens for other therapeutic options, laypersons grant the lowest value for gingival remodeling, as in the study by Rosa et al. [16]. 

Pinho et al. [18] obtained results with a distribution similar to that of Mota and Pinho [14]. In the last study, it was possible to observe a higher appreciation in the laypersons group than in the others. However, this difference was less marked in cases with tooth and gingival remodeling owing to a better ranking from dental professionals.

**Figure 5:**
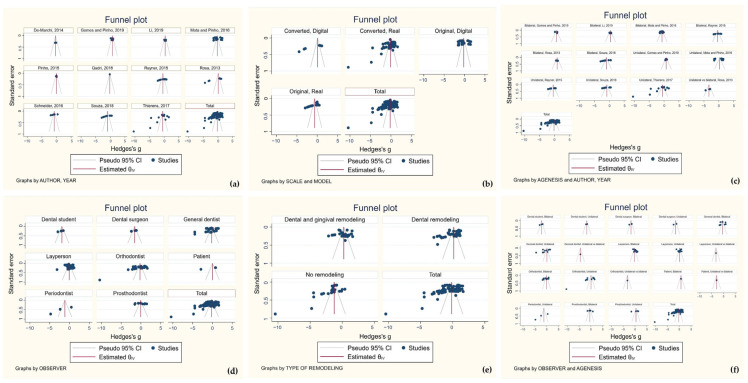
Funnel plot of publication bias by (**a**) author, (**b**) scale and model, (**c**) type of agenesis and author, (**d**) observer, (**e**) type of remodeling, and (**f**) observer and type of agenesis [7,9,14,16,18,25,48,50,51,52].

Regarding the sample used in each of the 11 studies suitable for the meta-analysis, as shown in Table 2, there was a disparity that may have induced an overestimation of the intervention effect, as suggested by the asymmetries shown in Figure 5a–f, and some amount of bias is possible. Two studies [16,52] had more publication bias than the other studies.

**Figure 6:**
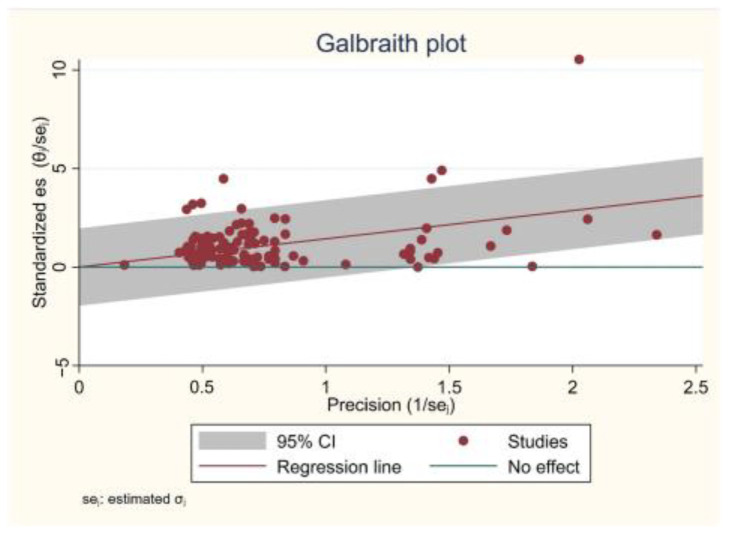
Heterogeneity assessment of effect sizes.

In Figure 6, heterogeneity among the effect sizes of the studies is suggested because several studies were outside the 95% CI region. Some studies, located toward the right of the x-axis, reported high precision. All studies were positioned on or above the green line, and the red line slopes slightly upward, indicating that the intervention is slightly more favorable than the control group.

In the paired *t*-test run on the type of treatment subgroup (space opening versus space closure) (Supplementary Table S2), the ideal image (6.93 ± 1.11) was valued more than the intervention (6.46 ± 1.09) with a significant decrease of 0.47 (95% CI, 0.7246 to 0.2126) *t*(13) = −3.99541, *p* < 0.002), which in this particular case revealed no preference for any type of treatment.

A paired *t*-test was performed on the type of remodeling subgroups (canine without remodeling, canine with dental remodeling, and canine with dental and gingival remodeling). Supplementary Table S3 showed no significant differences except for the subgroup “canine without remodeling” with the control image (ideal smile) (6.81 ± 1.29) being more valued than the no-remodeling image (4.47 ± 1.51) with a significant decrease of 2.34 (95% CI, 3.2270 to 1.4587), *t*(13) = −5.7245, *p*< 0.002), suggesting the need for remodeling in cases treated with space closure.

The results of the Shapiro–Wilk test of normality (α = 0.05; 95% CI) and boxplots for the identification of significant outliers (*p* < 0.05; 95% CI) enabled a valid paired *t*-test run (Supplementary Table S4 and Supplementary Figure S2).

## 4. Discussion

This review assessed the differences in esthetic perception between laypersons and dental professionals (those with specialized skills).

In light of the data obtained (*p* < 0.05), the null hypothesis that no differences existed among observers’ esthetic perceptions in different clinical situations of MLIA treatment was rejected. 

The authors followed a double strategy to include as many studies as possible. The search with the Boolean expression quickly limited the articles; the broad search validated the first, certifying that no research article was missing. Google Scholar is a free, easily accessible, and growing search engine and despite being more recent has the power to extract similar results [43] as other resources frequently used and reputable (Web of Science and Scopus). This strategy also provides insights into the scientific community’s interest in the subject. Nevertheless, despite its accuracy, it has few filters that retrieve a tremendous number of results, thereby producing a large amount of noisy data while searching. 

Globally, in the studies found, esthetic analysis does not follow standardized parameters as some studies [14,49,51] have considered both the sizes and characteristics of samples, whereas others [9,16] have considered only sizes. Both calculations were assessed during the methodological rating (Table 1), bearing in mind that the analysis of the first calculations [14,49,51] was more complete. Only five studies [9,14,16,49,51] performed sample size calculations. In one study [19] the authors admitted the lack of sample size calculation as a limitation, as the sample could not represent the entire population, assuming some bias. Two studies [11,53] were excluded for non-discrimination of the agenesis location or target population, single observer evaluation, or non-maxillary agenesis.

### 4.1. Differentiation Degree among Professionals

The studies did not accurately differentiate each professional category, mainly orthodontists, as official specialization is still being implemented in many countries. An orthodontist can be a professional with clinical experience in orthodontics but with unofficial training. Therefore, a sharp distinction between them and general dentists is lacking, possibly biasing the results. Orthodontists were absent in three studies [7,49,51] or included without specifying their specific training [16,18,19,25,48,50,52], whether they were orthodontic specialists or equivalents [9], or whether the designation extended to senior specialty students and hospital consultants [6]. Only one study [14] described orthodontists as professionals with at least 2 years of full-time training in orthodontics and more than 50% of their clinical practice in the area. Assuming that an orthodontist should be responsible for the treatment plan and decision to open or close the MLIA space, and that orthodontic procedures are often needed before gingival remodeling in situations of space closure, it is important to better differentiate these professionals in future studies. As the treatment should be multidisciplinary, some authors [14,49,52] included restorative dentists, periodontologists, or prosthodontists in their evaluations without specifying their expertise levels. 

### 4.2. Age and Gender of the Participating Population

Regarding the participating population, only two studies grouped the population according to age (25–60 years [16] and a mean age of 25 years [49]), which is a relevant factor [54], as older groups of laypeople tolerate more discrepancies in smile esthetics than younger groups, except for gingival exposure >6 mm during the smile (considered nonesthetic by all age groups). By contrast, the influence of gender on the esthetic perception of smiles is considered insignificant in the literature [30,48,49,54,55]. In this study, most studies only briefly described the participant’s gender, while the others omitted the subject. At most, by reading the results, we can say that there is a tendency for females to prefer narrower teeth and a greater step between the edge of the remodeled canine and the edge of the reference central incisor [48], and females tend to give higher scores [49], but differences may be highly culture-dependent [4].

### 4.3. Digital and Real Models

Both digital and real models have advantages and disadvantages. The main benefit of the digital model is the absence of confounding variables. Produced by computerized handling and performed from an initial 2D virtual image or a clinical photograph, it maintains the same teeth and lips, and therefore the same smile, introducing only slight variations. However, digital representations do not fully represent actual clinical situations in daily life, making it difficult to perceive the image and its appreciation [16]. The data obtained in this review show a tendency for higher ratings when this method is employed, because the images appear more perfect, which is a source of involuntary bias. A real model has the advantage of representing reality in images, similar to daily clinical situations, and it does not follow a standard or reference. In contrast, eliminating distraction variables due to individuality is impossible with different teeth, lips, and smiles. These natural features distract the viewer, biasing the evaluation with a tendency to identify more anatomical defects, which may justify the lower ratings. 

To minimize these differences, some studies have focused on the lower face, overlapping the same lips in different agenesis phenotypes [6,16,18]. In contrast, others digitally morphed a female model to represent the most prevalent type of that gender [9,14]. Studies that used real models [7,19,49,50] failed to eliminate confounding variables with an inherent evaluation bias. The digital manipulation of a real model with the elimination of confounding variables using the same lips, teeth, and face was formerly proposed [9] to allow a perception closer to reality through the real model. A similar methodology with minor digital manipulations over an original photograph to obtain a range of simulations has been identified [25,48,51,52]. 

A transversal constraint found in most studies is the restricted time for image observation (a few seconds), which allows only the observer’s first impression, probably biassing a score that could change with a more extended viewing period.

### 4.4. Rating Scales

The use of rating scales that were not directly comparable forced a fundamental convenience scale conversion but was a limitation. The VAS allows for a wider value choice, with the selection of values on a non-numeric reference scale with possible fractional values, which is unlikely with the 0–10 scale with only unique integers. This was the most relevant limitation of this systematic review, because it forced the adaptation of the results with no bibliographic references to support this conversion. In addition, two studies [49,50] used a scale of 0 to 5, which further strengthened the results. Another pertinent conversion was from the quantitative scale of the mean with standard deviation [7,14,16] or the median [9] to the mean with a 95% confidence interval, given in only one study [18]. However, this conversion is supported [44,45], allowing for the comparison of values in the same units of measurement, making the values comparable. Future studies could adopt a numeric rating scale (NRS) to standardize the evaluation of subjective perception of smile esthetics. VAS and NRS are concordant for evaluating both extra- and intra-oral images, are not influenced by differences between evaluators, and are simple methods; however, NRS is easier to deal with [56].

### 4.5. Smile Evaluation

The lack of significant differences (*p* < 0.05) between the two types of treatment found in the “Opening vs. Closure” group (Figure 2a,b and Supplementary Table S2) may be explained within each study by the inability of laypersons to detect subtle differences between situations, ranking both types of therapy with high scores [7,18]. However, in the inter-article classification, there was a discrepancy between the absolute results, which can be explained by the different scales and subsequent scale conversions. Nevertheless, the results indicated that both treatments achieved similar esthetic results (*p* < 0.05) [7,18]. In the group “No remodeling vs. Dental remodeling vs. Dental and gingival remodeling”- Unilateral (Figure 3a,b), the main differences existed between the presence or absence of symmetry, especially perceived by laypersons, to whom the most important factor is the symmetric morphology of the canine compared to the contralateral incisor when space closure is performed. Furthermore, mimicry between the lateral incisor and canine was valued more by laypersons than by dental professionals (*p* < 0.05). In contrast, the value of gingival remodeling was similar to that of isolated dental remodeling (*p* < 0.05). Paradoxically, for bilateral [9,16] and unilateral treatment [9], these authors found a reversal in the results obtained by laypersons and by the different groups of professionals, with laypersons scoring better images of “no remodeling” and “dental remodeling”, as seen more recently [51], a result not observed for images of “dental and gingival remodeling.” 

The greater ability to detect details and greater technical demands of professionals due to specific training could explain this reversal, or perhaps laypersons’ lack of perception of the changes in gingival margins may account for the reversal instead. Thus, gingival remodeling of canines should be weighed because it often requires supplementary orthodontic techniques, such as canine extrusion or premolar intrusion, gingival zenith change, and sometimes extensive coronary recontour [14], or even the need for mucogingival plastic surgery. Laypersons undervalue these procedures, and tolerate asymmetries of the gingival margin at the level of the central incisors up to 2 mm [9], the threshold for professionals being only 0.5 mm [9]. Given this fact and knowing that the asymmetries are more notorious closer to the midline, we can consider tolerable small asymmetries between the lateral incisors and canines [8]. To minimize differences in assessments, some authors [9,14] have used laypersons with advanced academic studies to approach the professional population. These differences remained, suggesting that professional training could be the primary key to valuing a smile. Regardless of the chosen treatment, if the agenesis is bilateral and attains a symmetrical treatment result, the result is accepted as esthetic, with higher global scores consistent with results from previous studies [8,18,34]. Therefore, one should seek symmetry in relation to the midline, knowing that, on average, orthodontists are more able to detect midline deviations exceeding 2 mm, while laypersons only notice variations greater than 3 mm [16,34].

### 4.6. Canine Morphology

Despite some limitations (canine esthetic variables changed separately and not as a whole and qualitative rating), one study [6] allowed us to understand how the width, height, morphology, color of the canine crown, and height of the gingival margin can alter the classification by itself. It was concluded that laypersons prefer narrower canines and value brighter hues than professionals. The existence of a positive correlation between the darkest canines and less attractive smiles, a fact recently highlighted [51,52], indicates that a simple change in the canine color hue makes smiles more attractive. A similar result was recently described in a study that focused on the perceptions of dental dyschromia according to patients and dentists, although it was not related to agenesis [57]. 

However, there is no consensus regarding the width of the canine as a substitute. Gomes and Pinho [25] partially contradicted two others [6,52], observing that all groups of observers preferred broader canines in a digitally manipulated specific clinical situation (asymmetric mesialized canines with differences in shape, color, and gingival contour). Notably, that study [25], despite having used a rule to distinguish canines as smaller or larger, had a ruler description that was somewhat confusing, without tangible value as a reference, preventing a more informed comparison between studies. Another study [52] also raised doubts about the width parameters as it used the original canine as an initial reference, raising doubts about matching these actual dimensions with those occurring in an average population. To overcome this, future studies should routinely use the canine width compared with the central incisor in the frontal view, as previously suggested [30,58]. Therefore, there is an urgent need for more extensive studies and randomized clinical trials. The divergence in these results may also be related to temporal changes in esthetic concepts or even to the geographic origins of perceivers, as has been suggested [59] since the participating populations were from different cultural roots, or the divergence may perhaps be related to the chosen substitute canine edge width or height [48], possibly affecting esthetics in the treatment of maxillary canine substitution. Additionally, the subjectivity of esthetic perception and possible changes based on the shared beliefs and standards of a specific community cannot be forgotten.

Li et al. [48] found that a canine with an edge width of 62.5% of the central incisor width and an edge height of 0.5 mm gingival to the central incisor edge was considered the most esthetic shape for the canine. Orthodontists do not appreciate marked cusp slopes (>1.0 mm) [48]. Simultaneously, laypersons preferred cusps between 1.0 and 1.5 mm. These results for laypersons have been reported [13] along with the lack of esthetic impact of the wear of the canine cusps, a detail that could favor a slight step between the edge of the substitute canine and the edge of the central incisor [48,60]. Recently, it was found [55] that when the lateral incisor was the mater, laypersons preferred wider teeth, with tendencies for measurements far beyond the 62.5% reference of the golden width/height proportion, the relationship most valued by orthodontists. 

This systematic review has revealed that standardized and randomized clinical trials are still needed to compare symmetrical MLIA space opening or closure and to evaluate asymmetrical situations with the need to use the same rating scale. Given the data obtained, dental professionals must abstain from giving their personal opinions when recommending treatment options for an MLIA situation because discrepancies exist between the treatment result judged as most esthetic and that most likely to be recommended.

### 4.7. Different Options for MLIA Management

Based on all the data collected, the management of maxillary lateral incisor agenesis in a growing young population can include (1) monitoring because in some cases, no treatment may be necessary if the missing tooth does not affect the patient’s dental health, function, or esthetics (in these cases, the mesial drift of the canine is allowed); (2) space maintenance to prevent the adjacent teeth from drifting into the empty space, preferably with a fixed tooth-shaped space maintainer until a permanent replacement tooth can be placed; and (3) orthodontic treatment depending on the severity of the misalignment, the planned closure or space opening, or other orthodontic issues. In young adults and adults, besides those options, there are two alternatives: (4) a single-tooth implant, preferably as late as possible to delay infraocclusion, or (5) tooth-supported restoration in the form of a dental bridge with one or two supporting wings.

## 5. Conclusions

The esthetic perception of MLIA treatment is controversial, even among professionals. Laypersons cannot value space opening versus space closure and value symmetry. Orthodontists are among the most demanding professionals in line with their expertise in the area. Gingival remodeling was not significantly more valued (*p* < 0.05) than isolated dental remodeling. In doubtful situations, a discussion with a less demanding patient is pertinent. Therefore, dentists should avoid overtreatment. Randomized clinical trials are still needed to compare symmetrical MLIA space opening or closure and to evaluate asymmetrical situations. Comparable rigorous methodologies, such as the standardization of the group of observers and rating scale, are needed.

## Figures and Tables

**Table 1:** Synthesis of qualitative analysis for risk-of-bias assessment.

	Sample Size Calculation	Selection Description	Dropout	Valid methodology	Confounding Variables	Blind Measurements	Adequate Statistics Analysis	Qualitative Scoring
Armbruster et al. 2005 [19]	-	?	+	+	-	+	+	Moderate
Brough et al. 2010 [6]	-	+	+	+	+	+	+	High
De-Marchi et al. 2014 [7]	-	?	+	+	-	+	+	Moderate
Gomes & Pinho 2019 [25]	-	+	+	+	+	+	+	High
Li et al. 2019 [48]	-	?	+	+	+	+	+	High
Mota & Pinho 2016 [14]	+	+	+	+	+	+	+	High
Pinho et al. 2015 [18]	-	?	+	+	+	-	+	Moderate
Qadri et al. 2016 [49]	+	+	+	+	+	-	+	High
Rayner et al. 2015 [9]	+	+	+	+	+	+	+	High
Rosa et al. 2013 [16]	+	?	+	+	+	+	+	High
Schneider et al. 2016 [50]	-	?	+	+	-	+	+	Moderate
Souza et al. 2018 [51]	+	+	+	+	+	+	+	High
Thierens et al. 2017 [52]	-	+	+	+	+	+	+	High

(+)—Item with quality; (?)—Item with dubious quality; (-)—Item without quality; Scored by number of (+) as High (5–7), Moderate (3–4), or Low (0–2) quality.

**Table 2:** Sample sizes according to author and observer.

Author, Year	Type of Observer	Sample
Armbruster et al. (2005) [19]	General dentist	140
	Layperson	60
	Orthodontist	40
	Dental specialists	29
Brough et al. (2010) [6]	General dentist	40
	Layperson	40
	Orthodontist	40
De-Marchi et al. (2014) [7]	Orthodontist	20
	Periodontists	20
Gomes and Pinho (2016) [25]	General dentist	141
	Layperson	142
	Orthodontist	100
	Periodontists	51
Li et al. (2019) [48]	Layperson	60
	Orthodontist	41
Mota and Pinho (2016) [14]	General dentist	215
	Layperson	303
	Orthodontist	81
	Prosthodontist	55
Pinho et al. (2015) [18]	General dentist	181
	Layperson	120
	Orthodontist	80
Qadri et al. (2016) [49]	Layperson	959
Rayner et al. (2015) [9]	General dentist	30
	Layperson	30
	Orthodontist	30
Rosa et al. (2013) [16]	General dentist	40
	Layperson	40
	Orthodontist	40
	Patient	40
Schneider et al. (2016) [50]	General dentist	100
	Orthodontist	87
Souza et al. (2018) [51]	Dental student	50
	Dental surgeon	50
	Layperson	50
Thierens et al. (2017) [52]	General dentist	77
	Layperson	46
	Orthodontist	37
	Periodontists	14

**Table 3:** Data extracted and collected for qualitative analysis from the studies included in the present systematic review (population, intervention, objectives, outcome, validity of the methodology, confounding variables, and study design).

Study	Population	Interventions	Objectives	Parameters	Outcome	Validity of Methods	Confounding Variables	Study Design
Armbruster et al. (2005) [19]	40 (L) 140 (GDPs) 43 (O) 29 specialists (SP) (9 PT, 11 END, 3 SUR, 4 OD, and 2 P)	Direct visual observation. Observers blinded for treatment options.	Esthetic appearance of various treatment options of treated MLIA cases.	MLIA treated with Maryland bridges (MB), dental implants (IP), orthodontic canine reposition (CR), or no-MLIA (control)(NT). 1 (more) to 5 (less attractive).	(O) NT > CR > MB > I (GDPs)-NT = CR > MB = I (SP)-NT = CR > MB = I (L)–CR > NT > MB > I (*p* < 0.001).	YES real model	YES multiple variables	N-RCT CI
Brough et al. (2010) [6]	40 (L) 40 (GDPs) 40 (O)	Direct visual ranking of images digitally manipulated from original photography. Blind and random evaluation.	Smile attractiveness in patients with MLIA vs. natural whole dentition.	Gradual increment of canine width, crown height and morphology, and gingival margin height. No quantitative measures.	(All) Dark, large canines, gingival margin >0.5 mm above central incisive-unattractive. Narrow canines–better rank. (GDPs)-natural tones; (O)-slightly brighter tones. (L)-brighter tones; (O)-cusps < 1.0 mm; (L)-cusps 1.0–1.5 mm.	YES digital model	NO same teeth same gum	N-RCT NCI
De-Marchi et al. (2014) [7]	20 (L) (10M,10F) and 20 (GDPs) > 4 years practice (10M,10F)	Direct visual observation of 68 photographs 26 (SC + R) 20 (SO + IP) 22 (no-MLIA).	Attractiveness of smiles in patients with MLIA vs. natural whole dentition.	Controlled photographic protocol. Unpleasant: 0 to 50.99 mm. Nice: 51–100 mm	(Male GDPs)-most critical Volunteers -control group–very pleased with their smiles. Patient satisfaction SC +R > SOI; more satisfied than control group (*p* < 0.002).	YES real model	YES different lips different teeth	N-RCT CI
Gomes & Pinho (2019) [25]	142 (L) 141 (GDPs) 100 (O) 51 (PT)	Quiz. Numerical Valuation. Ranking in ascending order. Anonymous.	Esthetic perception of asymmetric MLIA treated with SC and canine mesialization.	Space closure of MLIA with asymmetric canines. 2 symmetric simulations. Digital manipulation (smile 1—smaller canines) (smile 2—larger canines). Visual analog scale (VAS) (0–10).	Pretreatment image-least esthetic. Orthodontic treatment- improvement. Symmetric canines–most esthetic. Larger canines–more esthetic. Differences between (GDPs and L) regarding the most and least esthetic approach(*p* < 0.05). (L) more impressed than professionals; dental specialists more demanding (*p* < 0.05).	YES real model	NO same parameters same model	N-RTC NC
Li et al. (2019) [48]	60 (L) 41 (O)	Direct visual photo observation. Ranking of images digitally manipulated from original photography. 140	Canine edge width and height affect in dental esthetics in canine mesialization.	127 closure treatments. Top 5 most pleasant cases, digitally manipulated; 140 images with canine edge widths ((0, 12.5, 25, 37.5, 50, 62.5 and 75% of the central incisor width) and heights (−0.5, 0, 0.5 and 1.0 mm relative to central incisor edge)).	Most esthetic canine shape-canine edge-62.5% of the central incisor width and −0.5 mm gingival to the central incisor edge (*p* < 0.005). Canine edge width (*p* = 0.003) and height (*p* < 0.001) affect esthetics in canine substitutions.	YES Digital model	NO same gingiva same teeth	N-RTC NCI
Mota & Pinho (2016) [14]	303 (L) 215 (GDPs) 81 (O) 55 (PT)	Online survey. Digital manipulation.	Perception of smile attractiveness in MLIA cases treated with canine mesialization.	9 digital photos of MLIA treatment involving space closure. Unilateral and bilateral. Numeric scale (1–10) (least to most attractive) >5-attractive <5-unattractive.	(L)-better scored all cases/other groups. (All)–ideal smile = smile with lateral incisors. (All)–canine remodeling-more attractive. GDPs/O/PT- favor canine remodeling + gingival remodeling.	YES digital model	NO	N-RCT CI
Pinho et al. (2015) [18]	120 (L) 181 (GDPs) 80 (O)	Online survey. Esthetic perception preferences. Pre-and post-treatment evaluations.	Smile esthetic perception in patients with MLIA with respect to gingival exposure.	4 clinical cases. 24 smile photos. Numerical scale 0–10.	All photos score- O < GDPs < L. Males- highest scores. Symmetric cases and medium smile- higher scored. Gingival exposure- significant influence on the esthetic perception in post-treatment cases (*p* < 0.001).	YES real model	YES same lips different teeth	N-RCT NCI
Qadri et al. (2016) [49]	959 (DSt) and University staff F/M (76%/24%) 5 (O) 5 (RD)	Online survey. 959 completed responses with 9590 judgments. 4 pairs of photos. BILATERAL	Esthetic perception concerning the outcome of bilateral MLIA treatment patients with SC, SO, or IP.	21 patients (11 SC/10 PR). 10 specialist dentists (O + RD) ranked the photos. Most attractive (1)-least attractive (22). Only bilaterally MLIA included in this study.	SC-more attractive/PR (*p* < 0.001). Females and staff-higher ratings. Females/males-preferred SC/PR = 3/1 (*p* < 0.001). Space closure more attractive than space opening by (L).	YES Real morphed model Photo size standardization	YES multiple variables	N-RCT NCI Cross-sectional
Rayner et al. (2015) [9]	30 (L) 30 (GDPs) 30 (O)	Direct visual observation. Digital manipulation. (average female face image based on frontal photos of 4 female volunteers).	Effect of canine characteristics and symmetry on perceived smile attractiveness, in MLIA treated with canine mesialization.	1 ideal image. 6 morphed images (canine with lateral incisor-unilateral and bilateral). 3 types of canine created using software. Variations in shape, length, and color.	(O, GDPs)-space closure with canine significantly less attractive/ideal smile unless replaced by ideal canines(*p* < 0.001). (L)-lateral incisors replaced with canines different from ideal smile, but not clinically significant. (All)– unilateral replacement not significantly less attractive than bilateral replacement.	YES real model	NO same face same teeth same smile	N-RCT CI
Rosa et al. (2013) [16]	40 (L) 40 (OP) 40 (GDPs) 40 (O)	Quiz. Digital model of an ideal smile. Ranking (descriptive analysis). Numerical valuation.	Valuation of esthetic perception in altered smiles due to MLIA with or without treatment.	12 simulations. Visual analog scale (VAS) 0 to 100.	Significant differences–(All professionals) and (L) (*p* < 0.005). Orthodontic treatment, absence of diastema, symmetry-higher valued by all groups.	YES digital model	NO same parameters same model	N-RCT NCI
Schneider et al. (2016) [50]	100 (L) 100 (GDPs) 87 (O) Blinded observers	Direct visual photo observation. 9 frontal photos 3-SC + R 3-SO + IP 3-no-MLIA	Esthetic evaluation of implants vs canine substitution in patients with MLIA.	7 pairs of bipolar adjectives. Smiles classified from 1–5 (less-more attractive).	O/GDPs-no-MLIA more attractive than SC + R > SO + IP (Non-significant). L-SC + R > no-MLIA > SP + IP. L/GDPs-Better scores for SC + R. All groups-Worst scores for SO + IP (Nonsignificant).	YES Real Model Best photo preselection by orthodontists	YES multiple variables Mixed cases	N-RCT CI
Souza et al. (2018) [51]	(L) (GDPs) (DSt) 150 (22 and 40 y) Similar socioeconomic status	Direct visual observation. Digital manipulation. SC UNILATERAL BILATERAL (R + G) (R + B + G) ® (R + C + G) (R + B)	Perception of the attractiveness of MLIA replaced with canine mesialization.	Extraoral photograph. 20-year-old woman-normal occlusion. Software manipulation of original photograph. Mandibular arch without modifications. Various compositions with different sizes and proportions of height and width of the teeth to simulate repositioning of the canine on the left, right, or both sides. VAS 0 to 10, (less to more esthetic).	Original image–highest acceptance by (All). Lowest acceptance–left side alterations. Bilateral R + G-highest scores from (L). R + C-lowest score from (GDPs). (DSt)-least attractive–bilateral alterations Globally–(L)-lowest scores/other groups Least acceptable–(All) groups-bleaching (L)-attractive—bleached mesialized canines without treatment. (GDPs and DSt)- notice more differences than (L). (L)-cannot detect some interventions.	YES Real Digitally manipulated Model	NO same teeth same gum same mandibular teeth	N-RCT CI
Thierens et al. (2017) [52]	46 (L) 77 (GDPs) 37 (O) 14 (P) (age, experience, and gender, except the mean age of (O) to (L)) Female: Male (ratio 1.5:1)	Direct visual observation. Digital manipulation. Ranking by attractiveness. UNILATERAL	Size, morphology, and color of the substitute canine influence on dento-gingival attractiveness perceived by dental professionals and laypeople.	Standard image. Five series (width, color, gingival margin height, canine crown tip, and gingival margin height of the neighboring first premolar). Image most deviated from the standard/each parameter combined into a final series.	Dark canine and pronounced tip of a substituted canine-most unattractive to (All) professionals and (L). Gingival height of the neighboring premolar-least unattractive–(All) groups of examiners.	YES Real Digitally manipulated model	NO same teeth same gum same mandibular teeth	N-RCT CI

All-all groups of observers; B-bleaching; CI-control image; MLIA-maxillary lateral incisor agenesis; F-female; L-layperson; G-gingival recontouring; GDPs-general dental professional; M-male; NCI-no control image; N-RCT-non-randomized controlled trial; O-orthodontist; M-male; PT-prosthodontist; R-reshaping of the canine crown; SC + R-space closure and composite restorations; and SO + I-space opening + implant-supported crown.

## Supplementary Materials

The following supporting information can be downloaded at https://www.mdpi.com/article/10.3390/dj11040105/s1: Table S1: Difference in means and size effect calculation; Table S2: Paired *t*-test run on the type of treatment subgroup (space opening versus space closure); Table S3: Paired *t*-test run by type of remodeling (canine without remodeling, canine with dental remodeling, and canine with dental and gingival remodeling); Table S4: Shapiro-Wilk test of normality: (a) by type of treatment; (b) by type of re-modeling; Figure S1: Forest plots with differences in means, by author and: (a) type of remodeling, (b) type of agenesis, (c) observer; Figure S2: Boxplots for the identification of significant outliers: (a) Opening versus closure data; (b) Unilateral agenesis; (c) Bilateral agenesis.
