# Supplementary material for: Esthetic Perception of Different Clinical Situations of Maxillary Lateral Incisor Agenesis According to Populations with Dental and Non-Dental Backgrounds: A Systematic Review and Meta-Analysis
Source: Dent J (Basel). 2023 Apr 17;11(4):105. doi: 10.3390/dj11040105 (PMC10137431; doi:10.3390/dj11040105)
Supplement: Supplementary file 1 [file dentistry-11-00105-s001.zip › Supplementary Figure S1.docx]

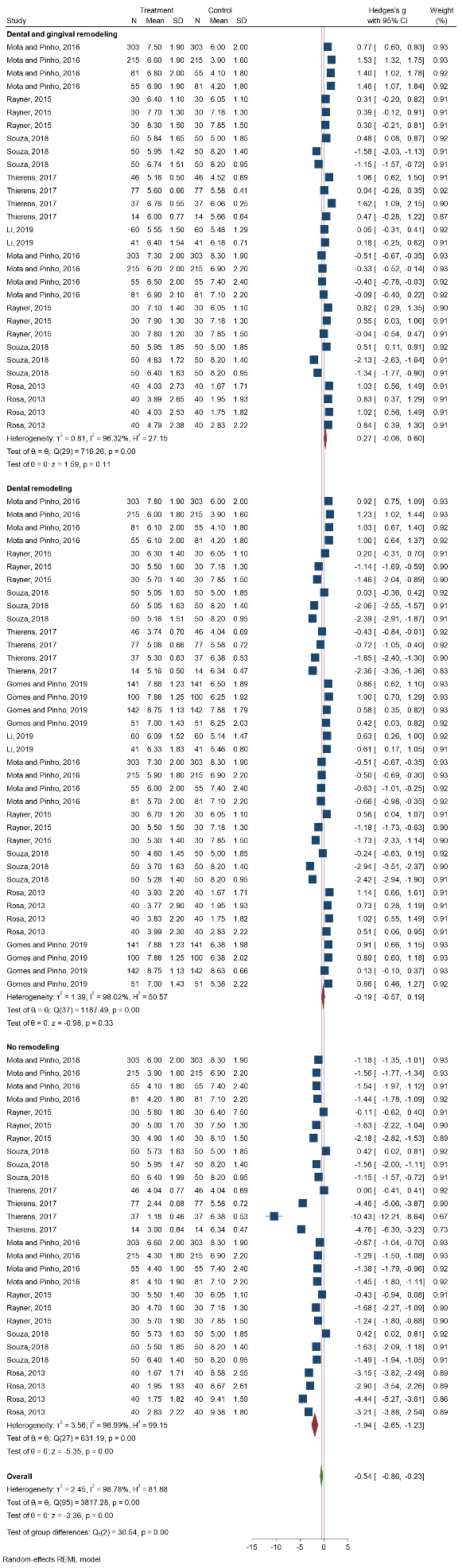

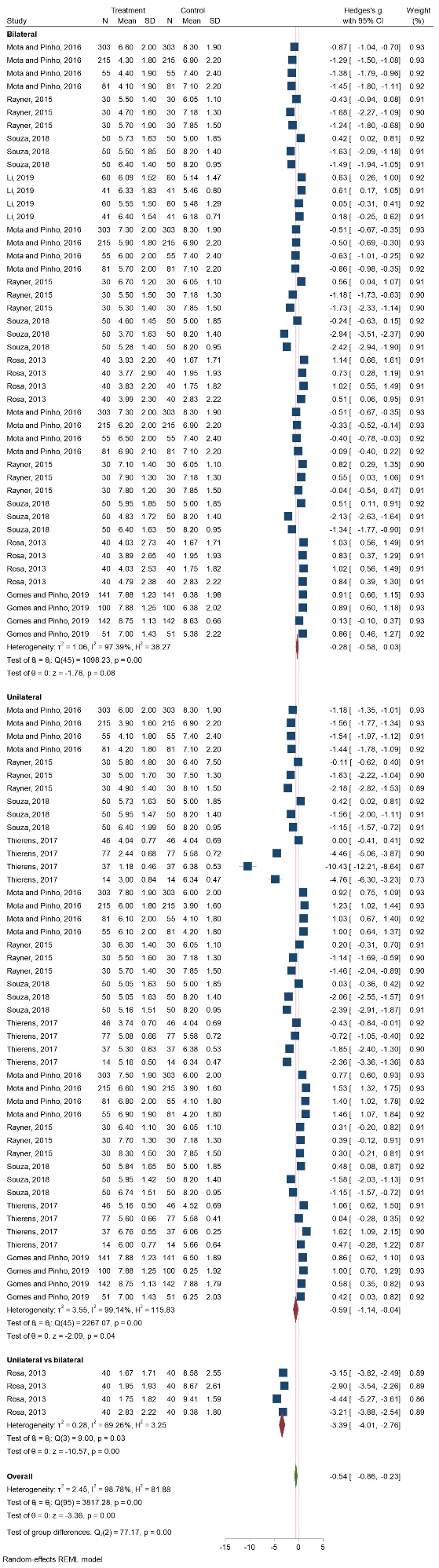

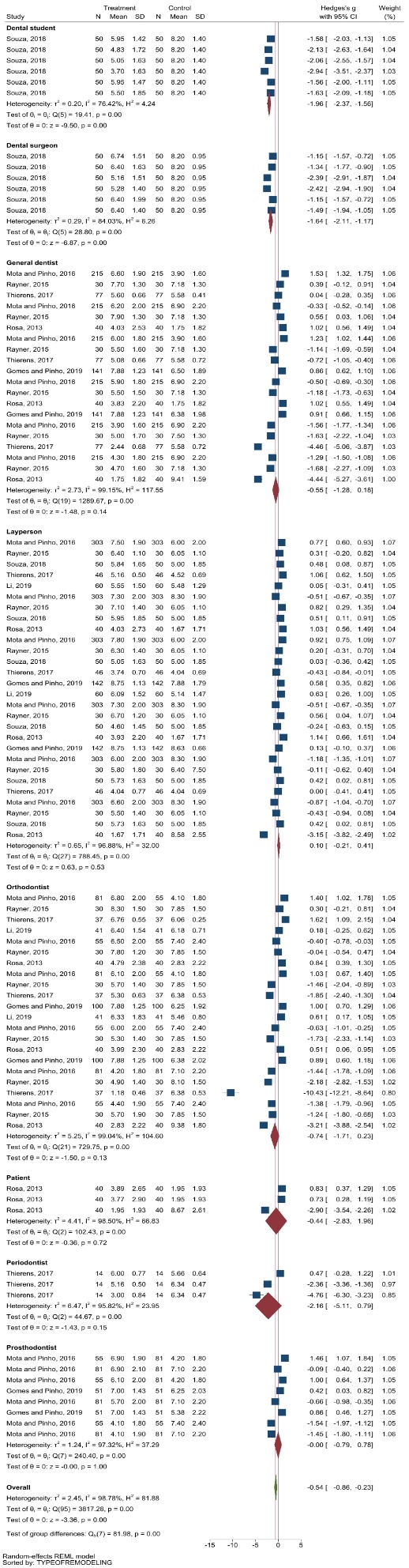


**(c)**

**(b)**

**(a)**

**Figure S1.** Forest plots with differences in means, by author and: (**a**) type of remodeling, (**b**) type of agenesis, (**c**) observer.
