# Supplementary material for: Esthetic Perception of Different Clinical Situations of Maxillary Lateral Incisor Agenesis According to Populations with Dental and Non-Dental Backgrounds: A Systematic Review and Meta-Analysis
Source: Dent J (Basel). 2023 Apr 17;11(4):105. doi: 10.3390/dj11040105 (PMC10137431; doi:10.3390/dj11040105)

**Supplementary Table S3.** Paired t-test run by type of remodeling (canine without remodeling, canine with dental remodeling, and canine with dental and gingival remodeling).


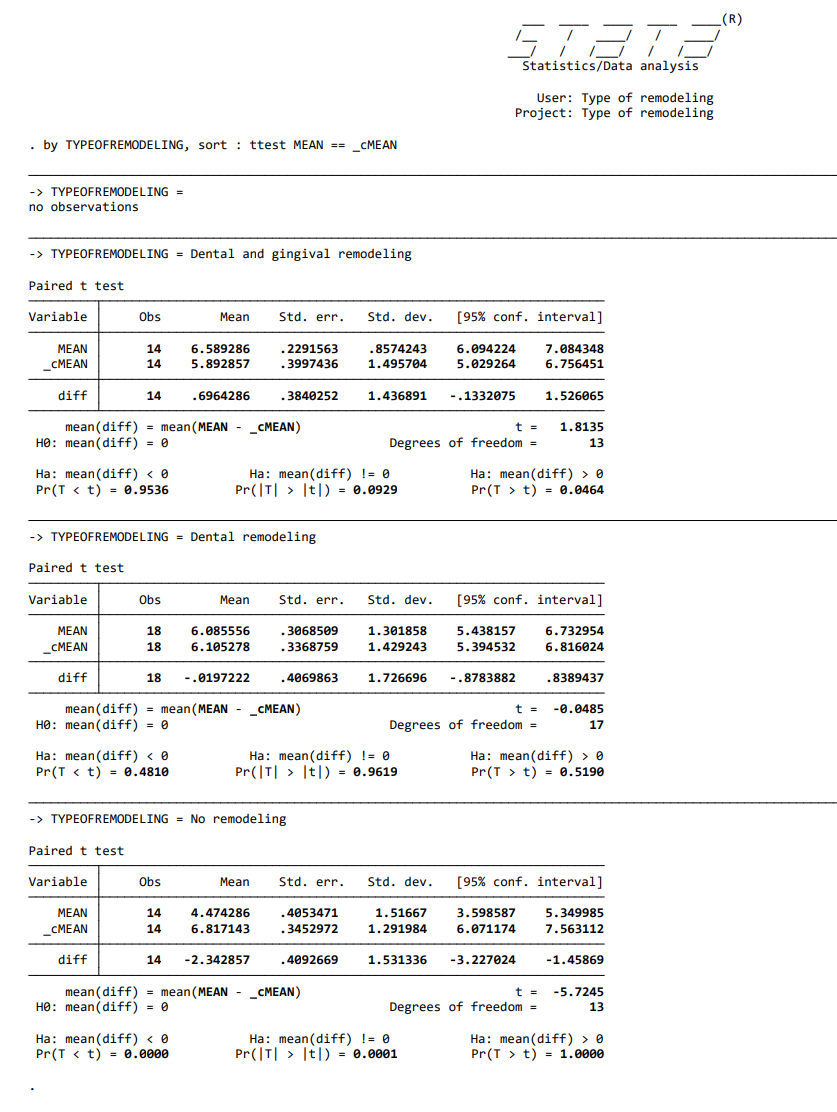

Supplement: Supplementary file 1 [file dentistry-11-00105-s001.zip › Supplementary Table S3.docx]
