# Supplementary material for: Esthetic Perception of Different Clinical Situations of Maxillary Lateral Incisor Agenesis According to Populations with Dental and Non-Dental Backgrounds: A Systematic Review and Meta-Analysis
Source: Dent J (Basel). 2023 Apr 17;11(4):105. doi: 10.3390/dj11040105 (PMC10137431; doi:10.3390/dj11040105)

**Supplementary Table S4.** Shapiro-Wilk test of normality: (**a**) by type of treatment; (**b**) by type of remodeling.

**(a)**


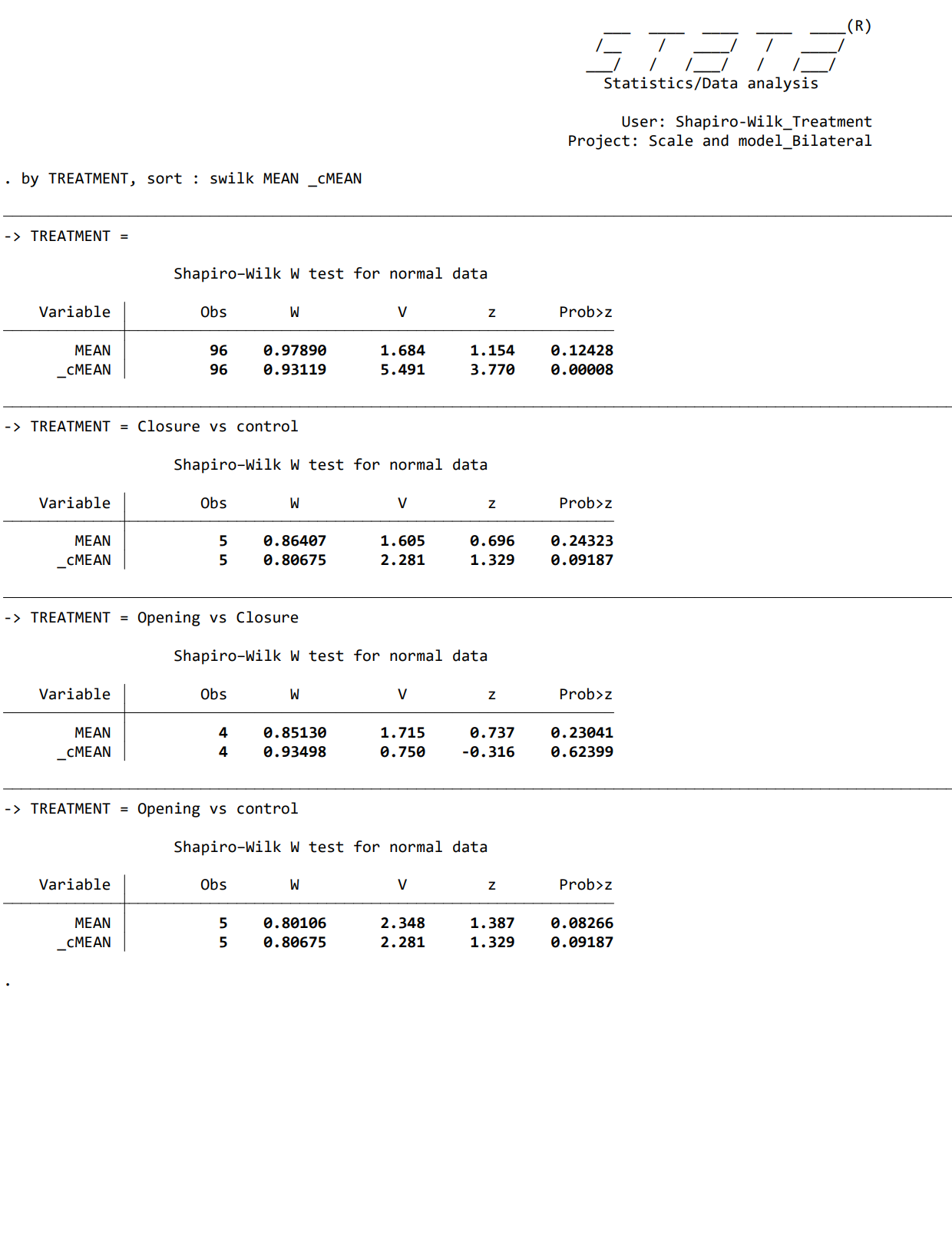


**(b)**


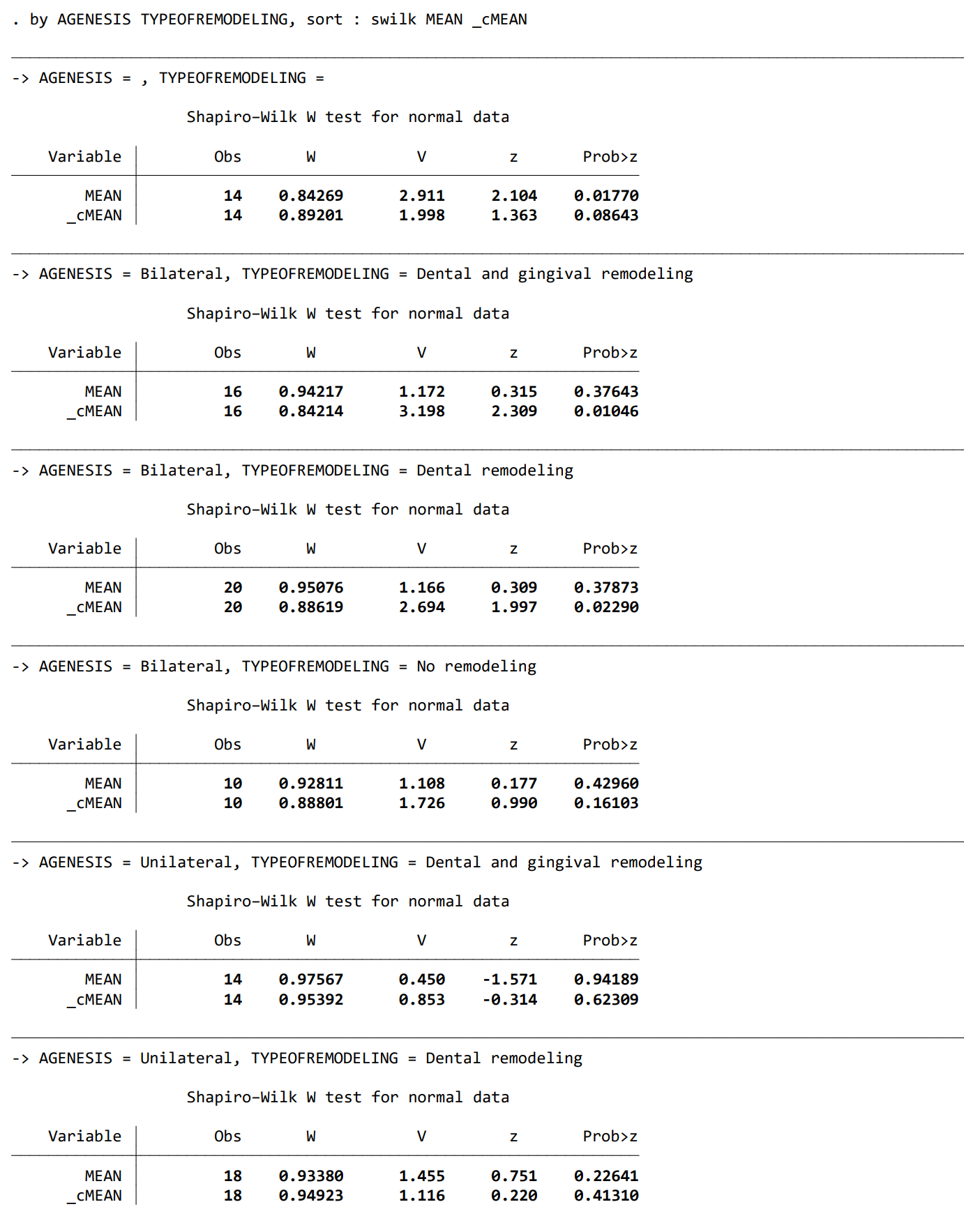


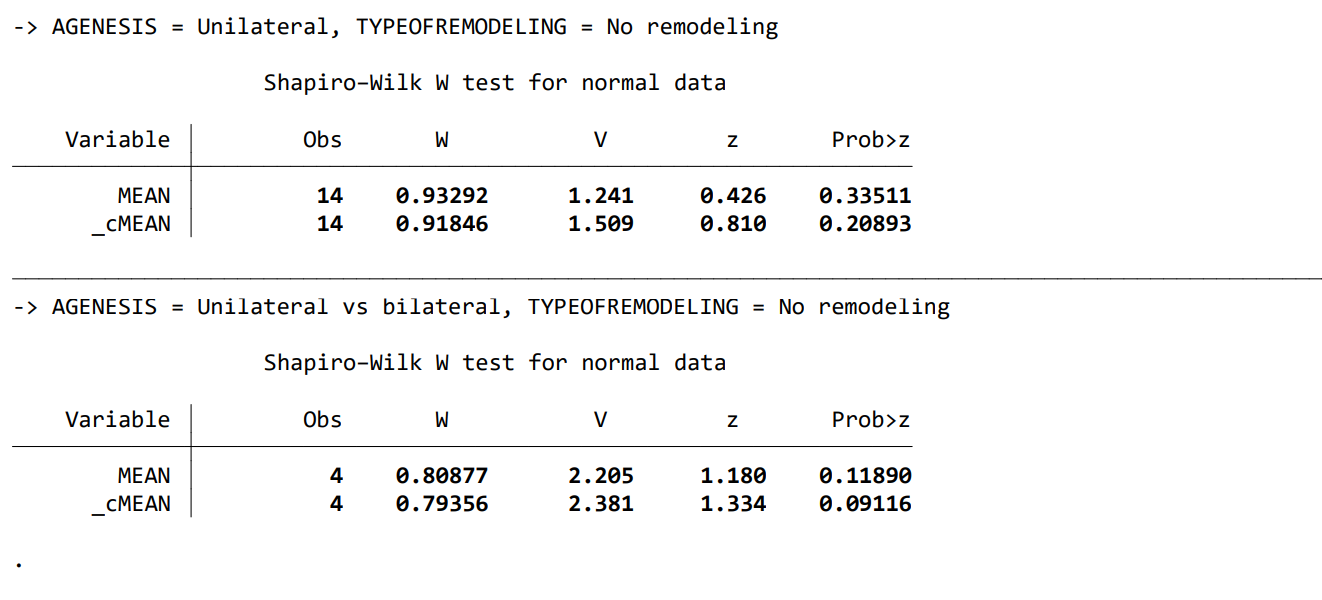

Supplement: Supplementary file 1 [file dentistry-11-00105-s001.zip › Supplementary Table S4.docx]
